# Validity and reliability of the HeartQoL questionnaire based on the EUROASPIRE IV study

**DOI:** 10.1186/2049-3258-73-S1-P8

**Published:** 2015-09-17

**Authors:** Delphine De Smedt, Els Clays, Stefan Höfer, Neil Oldridge, Kornelia Kotseva, Dirk De Bacquer

**Affiliations:** 1 Ghent University, Gent, Belgium; 2 Innsbruck Medical University, Innsbruck, Austria; 3 University if Wisconsin-Milwaukee, Wisconsin, United States of America; 4 Imperial College London, London, United Kingdom

## 

Coronary heart disease (CHD) is associated with a substantial physical and mental burden. Recently, HeartQoL, a core Health-related-quality-of-life (HRQL) instrument in patients with CHD, was developed. HeartQoL consists of 14 items; 10 items focusing on physical HRQL and 4 items on emotional HRQL together providing a global scale. The aim of the current study was to assess the reliability and validity of the instrument in a European sample of patients with CHD. Analyses are based on the recently performed EUROASPIRE IV (EUROpean Action on Secondary and Primary Prevention through Intervention to Reduce Events) survey (2012-2013). 7449 patients between 18 and 80 years old, with stable CHD who had been hospitalised for a first or recurrent coronary event completed the HeartQoL questionnaire. Psychometric analyses assessing the reliability and validity of the HeartQoL instrument were performed. The mean global score was 2.18 (0.66), the mean physical and emotional subscale scores were 2.13 (0.72) and 2.30 (0.72) respectively. Overall, excellent internal consistency was found on the global scale (a=0.92) and the physical subscale (a=0.91), and good internal consistency was seen on the emotional scale (a=0.87). Factor analyses confirmed the two-dimensional construct with factor loadings >0.5, with potential allocation problems on one item and fit indices which resulted in inconsistent outcomes. On country specific level, Bosnia scored poorly, probably due to a mistranslation of the questionnaire. Discriminative validity was confirmed with females reporting poorer global, physical and emotional scores, older patients reporting poorer global and physical scores and lower educated patients reporting poorer global, physical and emotional scores. Likewise convergent validity was confirmed with moderate to strong correlations among hypothesized constructs. Overall, psychometric analyses of the HeartQoL instrument in a population of patients with stable CHD showed good reliability and validity both at the European as well as on country-specific level.

